# Changes in Immunoglobulins G and A in the Saliva and Serum of Horses with Equine Gastric Ulcer Syndrome (EGUS) and Their Relationship with Other Immune and Redox Status Biomarkers

**DOI:** 10.3390/biology13110891

**Published:** 2024-11-01

**Authors:** María Botía, María Martín-Cuervo, Silvia Martínez-Subiela, José Joaquín Cerón, Ignacio Ayala, Sanni Hansen, Alberto Muñoz-Prieto

**Affiliations:** 1 Interdisciplinary Laboratory of Clinical Analysis of the University of Murcia (INTERLAB-UMU), Department of Animal Medicine and Surgery, Veterinary School, Regional Campus of International Excellence Mare Nostrum, University of Murcia, Espinardo, 30100 Murcia, Spain; maria.botiag@um.es (M.B.); silviams@um.es (S.M.-S.); jjceron@um.es (J.J.C.); iayape@um.es (I.A.); 2 Animal Medicine, Faculty of Veterinary Medicine of Cáceres, University of Extremadura, Av. de la Universidad S-N, 10005 Cáceres, Spain; mmcevet@hotmail.com; 3 Department of Veterinary Clinical Sciences, Veterinary School of Medicine, Sektion Medicine and Surgery, University of Copenhagen, Hoejbakkegaard Allé5, DK-2630 Høje-Taastrup, Denmark; sannih@sund.ku.dk

**Keywords:** horse, EGUS, saliva, IgG, IgA

## Abstract

Equine Gastric Ulcer Syndrome (EGUS) is a widespread disease with a very high prevalence and importance in horses. It includes two conditions: Equine squamous gastric disease (ESGD), linked to acid disturbances, and Equine Glandular Gastric Disease (EGGD), associated with inflammation and immune system issues. In saliva, increased levels of immunological markers have been observed in this disease, indicating immune system involvement. In addition, changes in biomarkers of oxidative stress have been described. In horses, IgG and IgA are proteins that appear in saliva and serum and are considered to be the major proteins in specific immunity; however, their levels in horses with EGUS have not been previously studied. This report aims to evaluate IgG and IgA concentrations in the saliva and serum of horses with EGUS, measured by automated assays, and compare them with other biomarkers of the immune and oxidative stress system. This work validated the IgG and IgA assays in saliva and showed that IgA significantly increased in the saliva of horses with EGUS. When the correlation between IgG and IgA and other biomarkers of immune system issues and oxidative stress such as ADA, S100 A8-A9 (calprotectin), S100 A12 (calgranulin), uric acid, FRAS, and AOPP was studied, IgA in saliva showed a significant moderate correlation with adenosine deaminase, indicating its possible involvement in the immune reaction occurring in EGUS.

## Simple Summary

Equine Gastric Ulcer Syndrome (EGUS) is a widespread disease. It shows variable clinical signs, such as behavioural changes, weight loss, and a reduced appetite, but it has a lack of obvious external symptoms and requires endoscopy for diagnosis. Saliva is gaining attention as a source of biomarkers for EGUS because it can show changes in its composition in response to this disease, and it is also easy and non-stressful to collect. Immunoglobulins (Igs) are important for detecting immune system dysfunctions. In this study, high concentrations of IgA in saliva were detected in horses with EGUS compared to healthy ones, and these changes correlated with other biomarkers from the immune system, such as adenosine deaminase. This would be in line with the results reported in humans, where IgA plays a role in mucosal protection and is elevated in gastric ulcers. These results indicate an involvement of the immune system in the development of EGUS in horses and open up the potential to use IgA as a possible biomarker of this disease.

## 1. Introduction

Equine Gastric Ulcer Syndrome (EGUS) is an extended worldwide disease with a high incidence and affects all breeds and types of horses [1]. The European College of Equine Internal Medicine (ECEIM) differentiates between two different diseases within this syndrome in its guidelines. Equine Squamous Gastric Disease (ESGD) is related to acid disturbances and affects the squamous mucosa, and Equine Glandular Gastric Disease (EGGD) is related to inflammation and immune system impairment affecting the glandular mucosa [2]. EGUS has variable clinical signs such as changes in behaviour, loss of weight, and a reduced appetite, and in many cases, it does not produce external evident clinical signs, being diagnosed by the presence of lesions at gastric mucosae detected by an endoscopy [3].

Saliva is gaining attention as a source of possible biomarkers of EGUS. Two biomarkers related to the immune system that can be detected in saliva are adenosine deaminase (ADA) and S100A8/A9. ADA is an enzyme involved in purine metabolism that plays a crucial role in the regulation of immune function, particularly in T-cell activation and proliferation [4]. S100A8/A9 is a heterodimeric protein complex involved in the regulation of inflammatory responses and immune system activation. It serves as a key biomarker for inflammation and immune cell recruitment, playing an important role in modulating innate immunity, and is often elevated in various inflammatory and immune-related conditions, being particularly associated with gastrointestinal diseases [5]. Both S100A8/A9 and ADA have been described to increase in horses with EGUS [6,7], indicating a possible involvement of the immune system in this disease. In addition, other analytes involved in other physiopathological pathways that may be related to inflammation have been described to be changed in this disease], such as uric acid, a molecule with antioxidant properties that, in excess, it can trigger the production of cytokines and chemokines, which intensifies inflammation and leads to endothelial dysfunction and fibrosis [8]; the ferric-reducing ability of saliva (FRAS), which indicates overall antioxidant capacity by evaluating the ability of non-enzymatic antioxidants in saliva to convert ferric-tripyridyltriazine into its ferrous form [9]; or advanced oxidation protein products (AOPPs) formed during oxidative stress by the action of chlorinated oxidants, mainly hypochlorous acid and chloramines on albumin [10]. Those analytes were correlated with ADA [11].

Measurements of immunoglobulins (Igs) are of interest to detect deficiencies or dysfunctions in the humoral immune system, situations in which Igs decrease, as well as increases in Igs, especially IgG, that occur in immune-mediated disorders or chronic diseases [12]. In previous reports in humans, the role of IgA in the immunological mechanisms involving mucosal protection and autoimmunity in ulceration processes in the stomach has been described. However, there is a controversy regarding the IgA response in serum in gastric ulcers in humans, since in one report, although non-significant, there was a tendency of IgA to decrease in these patients [13]. However, in another study, IgA was increased in the serum of patients with gastric ulcers [14]. In addition, IgA increases in the saliva of children with gastric ulcers due to *Helicobacter pylori* [15].

IgG and IgA can be detected in the saliva of both humans and animals, but their sources differ. Salivary IgG is primarily derived from blood, whereas IgA is mainly produced by the salivary glands in plasmatic cells. Interestingly, the IgA concentration can be influenced by factors such as stress, whereas IgG in saliva is more closely related to their concentration in serum [16]. In horses, IgG and IgA have been measured in saliva and are considered the major proteins of specific immunity [17]. However, to our knowledge, there are no reports that have studied IgG and IgA in the saliva or serum of horses with EGUS.

The objective of this report was to evaluate the concentrations of IgG and IgA in saliva and serum in horses with EGUS. For this purpose, two commercially available automated assays were validated from an analytical point of view and applied for the measurements of these analytes. In addition, IgG and IgA were compared with other biomarkers of the immune system, such as ADA, S100 A8-A9 (calprotectin), and other proteins of the S100 family, such as S100 A12 (calgranulin), as well as biomarkers of oxidative stress, such as uric acid, FRAS, and AOPP in order to gain knowledge about the possible relations between these analytes.

## 2. Materials and Methods

### 2.1. Horse Population

This study involved saliva samples from horses from the Large Animal Teaching Hospital at the University of Copenhagen and the Veterinary Teaching Hospital at the University of Extremadura, covering the period from August 2020 to August 2023.

All horses underwent a comprehensive clinical assessment, which included a detailed medical history and a physical examination. The physical examination encompassed weight assessment, body condition score (BCS) measured on a nine-point scale [18], an evaluation of heart and respiratory rates, rectal temperature, the colour of mucous membranes, capillary refill time, and borborygmus. Additionally, hematological and biochemical analyses were performed. Following a 16 h fasting period, gastroscopy was conducted on all subjects using previously established protocols [6].

Gastroscopy images were interpreted by a single experienced veterinarian at each hospital, both with over ten years of experience, according to the guidelines provided by the European College of Equine Internal Medicine (ECEIM) in its Consensus Statement [2]. A horse was diagnosed with ESGD if it had a score higher than 1 on the ESGD grading scale developed by the ECEIM Consensus, which ranges from 0 to 4 points. Similarly, a horse was diagnosed with EGGD if it scored higher than 1 on the same 0-to-4-point scale as previously described [19]. Additional diagnostic procedures, such as a rectal exam, abdominal imaging, abdominal centesis, or exploratory laparotomy, were performed as necessary.

Based on the outcomes of the clinical evaluations and diagnostic tests, horses were categorized into three distinct groups:

- EGUS group: They displayed clinical symptoms and gastroscopy images consistent with EGUS, according to previously established criteria [2]. This group was further divided into ESGD, EGGD, or both ESGD and EGGD (EGGD + ESGD). Only horses diagnosed with EGUS and without evidence of other illnesses were included in this group.
- Non-EGUS group: Horses showing clinical signs compatible with EGUS but lacking gastroscopy images indicative of EGUS were integrated into this group. The reasons for the gastroscopy of the horses are described in the Supplementary Table S1.
- Healthy group: These animals exhibited no clinical signs of abdominal pain or any other abnormalities during the physical examination; their hematological and biochemical results were within normal ranges, and they showed no signs of EGUS after the gastroscopy examination. This group only included horses from the University of Extremadura. These horses were submitted for castration, or OPU (Ovum Pick Up) procedures and a gastroscopy was part of their routine pre-surgical protocol.

A total of 105 horses were included in the study. The EGUS group consisted of 71 horses of different breeds, in which 25 horses had both types of EGUS (EGGD + ESGD) (9 mares and 16 geldings) with a median age of 11.28 years (range: 4–19) and a median BCS of 5.57 (range: 4–7). Within this EGUS group, 25 horses presented with only EGGD (8 mares and 17 geldings) with a median age of 11.04 years (range: 4–19) and a median BCS of 5.84 (range: 2–9), and 21 horses were diagnosed with ESGD (10 mares and 11 geldings), with a median age of 12.09 years (range: 6–24), and a median BCS of 5.51 (range: 3–7).

The non-EGUS group consisted of horses that presented with suspicious EGUS symptoms but had no compatible gastroscopy images and included 20 animals (13 mares and 7 geldings) with a median age of 11.8 years (range: 2–20) and a median BCS of 5.5 (range: 4–7). The suspicions that encouraged the use of gastroscopy in these horses are indicated in Supplementary Table S1.

The healthy group consisted of 14 animals (11 mares and 3 geldings) with a median age of 13.26 years (range: 5–23), a median BCS of 6.86 (range: 6–8) and was composed of different breeds.

There were no statistically significant differences in age and BCS between the groups.

### 2.2. Saliva and Serum Collection

Saliva and serum samples were collected from all horses before intravenous sedation, and the gastroscopy was performed immediately after the horses were positioned in the examination stand. Saliva was gathered using a sponge, which was then placed into a Salivette tube. These tubes were maintained at 4 °C and transported to the laboratory within 20 min of collection. Upon arrival, the samples were centrifuged at 3000× *g* for 10 min to extract the saliva, which was then stored at −80 °C until analysis. After collecting saliva samples, 5 mL of blood was drawn via jugular venipuncture and transferred into tubes (Becton Dickinson Vacutainer Systems Europe). The tubes contained a clot activator for serum collection used in routine biochemical analysis.

### 2.3. Measurement of Immunoglobulins and Analytical Validation in Horse Serum and Saliva

Salivary and serum IgG and IgA levels were measured using commercial kits designed for humans (IgG: Code number OSR6145, Olympus Europe GmbH; and IgA: Code number OSR1171, Olympus Europe GmbH) run on an automated analyzer (Olympus AU400, Olympus Europe GmbH, Hamburg, Germany) following the instructions of the manufacturer. The IgG assay was calibrated using a purified horse IgG material (Purified horse IgG, Forfis Life Sciences, Waltham, MA, USA), while IgA was calibrated with its own specific calibrator provided by the manufacturer (General Protein Calibrator, Spinreact S.A., Girona, Spain).

The IgG and IgA assays were validated for the equine saliva and serum samples using aliquots of samples collected from healthy horses and horses with different degrees of EGUS. The validation process for the assays was conducted as follows:

- Precision: Evaluated directly by calculating the intra- and inter-assay coefficients of variation (CVs) using samples with high and low concentrations of IgG and IgA.
- Accuracy: Assessed through a spiking recovery study in the case of IgG by mixing purified horse IgG material with a sample with a known concentration of IgG in the following proportions: 50–50%, 25–75%, and 75–25%. To calculate the recovery percentage, the observed results were divided by the expected results, and the value of this division was multiplied by 100. In the case of IgA, due to the lack of standard purified material, accuracy was indirectly evaluated by diluting a sample with a high concentration using ultrapure water.
- Limit of Detection (LoD): Defined as the lowest concentration of IgG and IgA that the assays could distinguish from a sample with zero value (ultrapure water), calculated by taking the mean value plus three standard deviations from 12 replicate measurements of ultrapure water.

### 2.4. Other Biochemical Analysis

The biochemical profile included in the saliva and serum samples was analyzed using an Olympus AU400 biochemical analyzer (Beckman Coulter Inc., Fullerton, CA, USA):

- Immune biomarkers: Salivary and serum adenosine deaminase (ADA) levels were measured with a commercial kit (ADA-D assay kit, Diazyme Laboratories, Poway, CA, USA). Salivary and serum S100 A8-A9 levels were measured with the BÜHLMANN fCal Turbo^®^ assay (BÜHLMANN, Laboratories AG, Schönenbuch, Switzerland). Salivary and serum S100-A12 levels were measured with a commercially available immunoassay (MBS008968 Horse S100 Calcium Binding Protein A12, S100A12, Mybiosource, San Diego, CA, USA) ELISA Kit.
- Redox biomarkers: Uric acid (UA) was measured using a colorimetric commercial kit (Uric acid, Beckman Coulter Inc., Fullerton, CA, USA). The ferric-reducing activity of saliva (FRAS) and the advanced oxidation protein products (AOPP) were measured following previously published methods [9,20].

Previous research has validated all these salivary assays for the immune and redox biomarkers [6,11,21,22] except for S100A12, which showed an inter- and intra-assay imprecision lower than 15% and recoveries rates in the 80–120% range in saliva and serum.

### 2.5. Changes in Immunoglobulins G and A in the Saliva and Serum of Horses with Equine Gastric Ulcer Syndrome (EGUS) and Comparison with Other Immune and Redox Markers

The concentrations of IgG and IgA were measured in the saliva and serum of all the horses in the study. In addition, immune and redox biomarkers were also measured, and correlations between these results and immunoglobulin concentrations were evaluated.

### 2.6. Statistical Analysis

Data were evaluated for normality using the Kolmogorov–Smirnov test, which indicated a non-parametric distribution. Consequently, a non-parametric test was chosen for comparative analysis. Intra-and inter-assay CVs, the LoD, and the CVs of the assays were calculated using Excel 2020 (Microsoft Corp., Redmond, WA, USA).

The Kruskal–Wallis test, followed by Dunn’s test for multiple comparisons, was employed to compare the medians among the different EGUS groups, including the controls. Correlations between different analytes and fluids were studied through the Spearman test. The degree of correlation was categorized based on the Spearman R-value, with 0.90 to 1 being indicative of a very high correlation, 0.70 to 0.90 indicating a high correlation, 0.50 to 0.70 indicating a moderate correlation, 0.30 to 0.50 indicating a low correlation, and less than 0.30 indicating little if any correlation. The results were presented as the median and interquartile range (IQR), with a significance level of *p* < 0.05. These analyses were conducted with GraphPad Prism 9 (version 9.5) (GraphPad Software, Boston, MA, USA).

The required sample size necessary to reach the statistical power of 0.8 was calculated using the following parameters: a significance level (α) of 0.05, the means and standard deviations of each group. These computations were carried out using G*Power software (version 3.1).

## 3. Results

### 3.1. Analytical Validation of Immunoglobulins G and A in Horse Saliva

**Figure 1:**
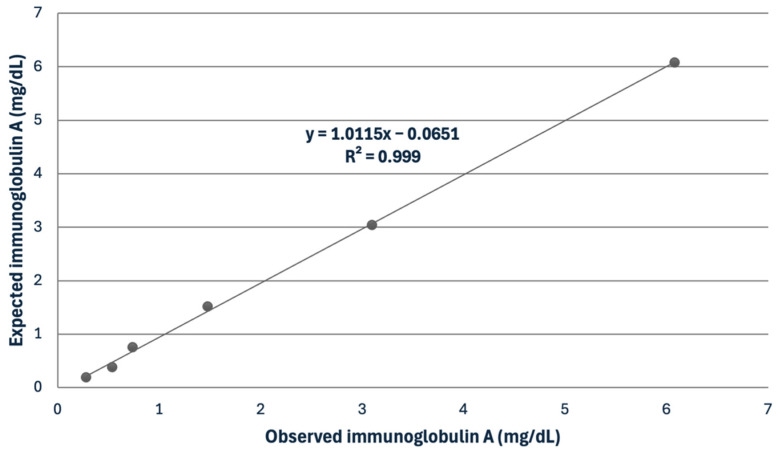
Linearity under dilution of a sample with a high concentration of immunoglobulin A in horse saliva.

In the saliva, the IgG assay showed mean intra- and inter-assay CVs of 5.12 and 7.73%, while the IgA assay displayed mean intra- and inter-assay CVs of 4.56 and 8.61% (Table 1). The recovery study for IgG showed recovery percentages ranging from 106.2 to 116.1%. The serial dilution of a saliva sample displayed a linear regression equation, exhibiting a coefficient of correlation approaching 1 for IgA (Figure 1). The LD was set at 0.6 and 0.44 mg/dL for IgG and IgA, respectively.

**Figure 2:**
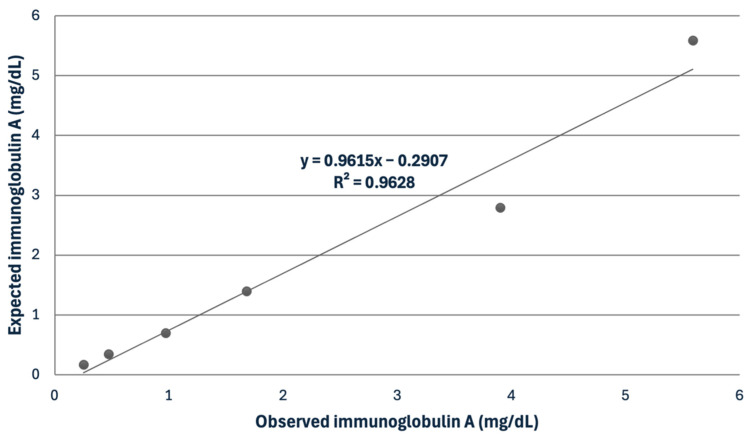
Linearity under dilution of a sample with a high concentration of immunoglobulin A in horse serum.

For the serum, the IgG assay showed mean intra- and inter-assay CVs of 3.47 and 6.53%, while the IgA assay had mean intra- and inter-assay CVs of 5.3 and 9.31% (Table 2). The recovery study for IgG showed recovery percentages ranging from 102.4 to 114.3%. The serial dilution of a serum sample indicated a linear regression equation, exhibiting a coefficient of correlation close to 1 in the IgA assay (Figure 2).

### 3.2. Changes in Immunoglobulins G and A in the Saliva and Serum of Horses with Equine Gastric Ulcer Syndrome (EGUS)

**Figure 3:**
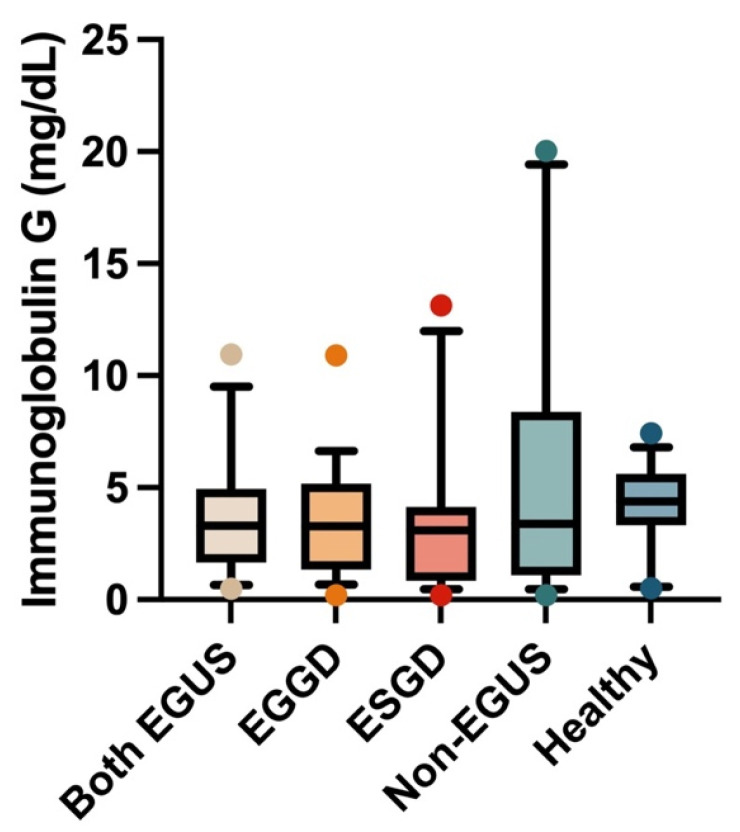
Concentrations of immunoglobulin G in the saliva of horses with both Equine Glandular Gastric Disease (EGGD) and Equine Glandular Gastric Disease (ESGD), horses with only EGGD, horses with only ESGD, horses presenting with clinical signs compatible with EGUS but without gastroscopy images compatible with EGUS (non-EGUS), and healthy horses. Horizontal lines represent the median concentrations, the boxes indicate the 10–90 percentiles, and the whiskers represent the ranges.

**Figure 4:**
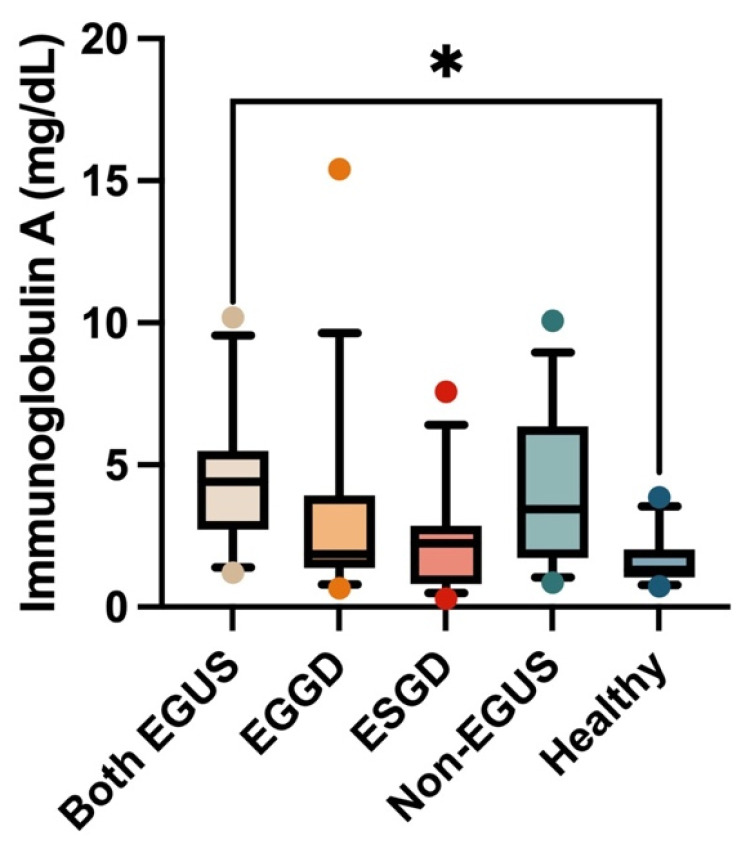
Concentrations of immunoglobulin A in the saliva of horses with both Equine Glandular Gastric Disease (EGGD) and Equine Glandular Gastric Disease (ESGD), horses with only EGGD, horses with only ESGD, horses presenting with clinical signs compatible with EGUS but without gastroscopy images compatible with EGUS (non-EGUS), and healthy horses. Horizontal lines represent the median concentrations, the boxes indicate the 10–90 percentiles, and the whiskers represent the ranges. * *p* < 0.05.

In the case of saliva, the IgG concentrations did not show significant variations in the different groups included in the study (Figure 3). However, a significant increase in salivary IgA concentrations was observed in the group of horses with both types of EGUS (median = 4.41 mg/dL, IQR = 2.73–5.49) compared to healthy horses (median = 1.3 mg/dL, IQR = 1.04–2.03) (*p* = 0.009) (Figure 4).

**Figure 5:**
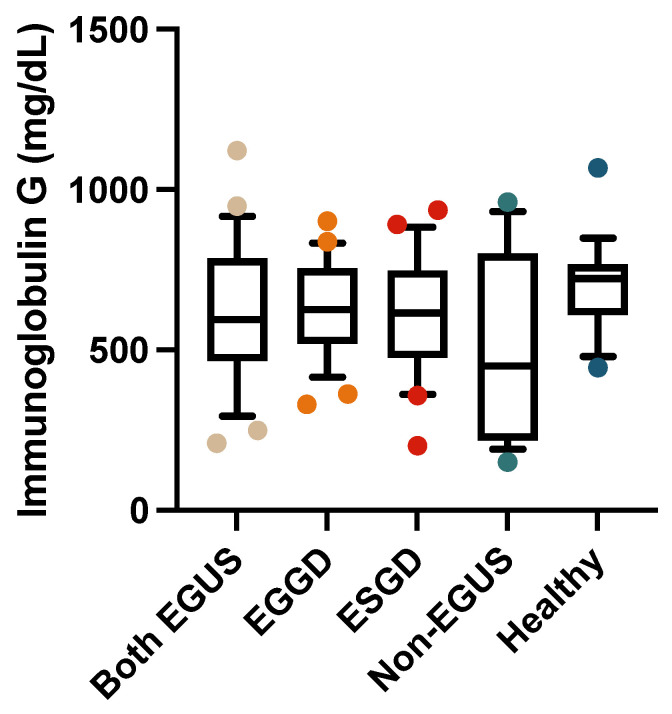
Concentrations of immunoglobulin G in the serum of horses with both Equine Glandular Gastric Disease (EGGD) and Equine Glandular Gastric Disease (ESGD), horses with only EGGD, horses with only ESGD, horses presenting with clinical signs compatible with EGUS but without gastroscopy images compatible with EGUS (non-EGUS), and healthy horses. Horizontal lines represent the median concentrations, the boxes indicate the 10–90 percentiles, and the whiskers represent the ranges.

**Figure 6:**
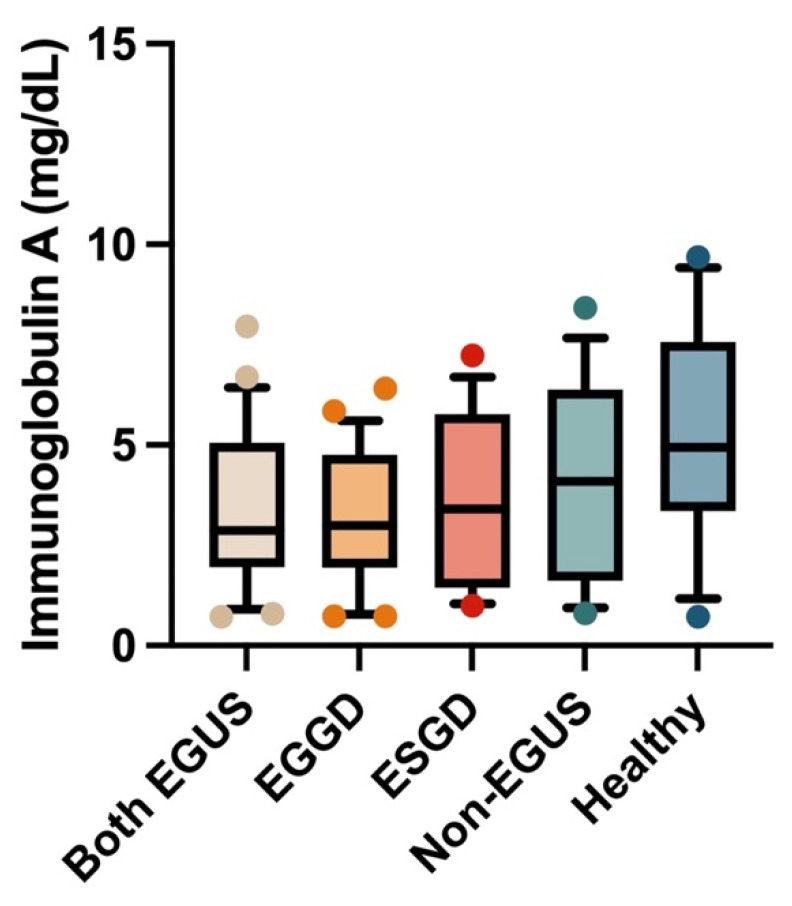
Concentrations of immunoglobulin A in the serum of horses with both Equine Glandular Gastric Disease (EGGD) and Equine Glandular Gastric Disease (ESGD), horses with only EGGD, horses with only ESGD, horses presenting with clinical signs compatible with EGUS but without gastroscopy images compatible with EGUS (non-EGUS), and healthy horses. Horizontal lines represent the median concentrations, the boxes indicate the 10–90 percentiles, and the whiskers represent the ranges.

In the serum, the concentrations of IgG or IgA did not show significant variations between any of the study groups (Figure 5 and Figure 6); however, the healthy horses showed a tendency to have higher concentrations of both immunoglobulins than horses with EGUS.

### 3.3. Changes in Other Saliva and Serum Biomarkers Related to Immune System and Redox Status in Horses with Equine Gastric Ulcer Syndrome (EGUS)

Table 3 shows the results for the other biomarkers related to the immune system and redox status measured in the saliva of horses with EGUS. In the case of ADA, higher activities were observed in all the EGUS groups compared to the healthy group. The levels of calprotectin, S100A12, and uric acid were significantly higher in all groups, including the non-EGUS group, compared with healthy horses. In addition, FRAS increased in all groups of horses with EGUS (EGGD + ESGD, EGGD, and ESGD) compared with healthy horses. Also, higher values were shown in horses with both types of EGUS than in the non-EGUS group.

The results for the other biomarkers related to the immune system and redox status in serum are depicted in Table 4. Uric acid and calprotectin showed decreased concentrations in horses with EGUS compared with healthy horses. Calprotectin also showed a significant decrease in the serum of the non-EGUS group compared with the healthy group.

### 3.4. Correlation Study of Immunoglobulins and Other Salivary and Serum Biomarkers in Horses

Salivary IgG showed low positive correlations with calprotectin (r = 0.35) and FRAS (r = 0.421).). IgA showed a low positive correlation with IgG (r = 0.376), ADA (r = 0.4), and calprotectin (r = 0.35).

For serum, IgA showed a high positive correlation with ADA (r = 0.762), and moderate correlations with FRAS (r = 0.553), calprotectin (r = 0.53), and uric acid (r = 0.53), while IgG did not show any correlation with any other biomarker.

No correlations were observed when comparing each biomarker between serum and saliva. The complete matrix of correlation is included in Supplementary Table S2.

## 4. Discussion

In this report, two commercially available automated assays for the measurement of IgG and IgA in saliva and serum were validated and applied to evaluate the possible changes in these analytes in EGUS, a disease with a high prevalence in horses.

The validated assays have the advantage of automation, although they could potentially be adapted to other formats, such as ELISA plates. The assay for IgG was calibrated with horse-purified IgG, and although its values are in the lower dynamic range of the method in the case of saliva, the assay was precise and accurate in both the serum and saliva samples. Similarly, the assay for IgA showed an imprecision lower than 15%, which is within the limit allowed [23] and was linear after serial sample dilution. Ideally, purified IgA from horses should have been used as a calibrator for the IgA assay; however, it is not currently commercially available [17]. Although the assays used were designed for the measurement of human Igs, they showed reactivity with the horse samples, in line with previous reports in which antibodies against human Igs reacted with horse Igs [24], being this is an example of the possible use of heterologous assays for the measurement of proteins [25].

The assays used in our report provided values of IgG and IgA in saliva that were in the range of those described in other studies performed using the saliva of horses, which reported values ranging from 0.6 to 128 mg/L for IgG (in our study, the salivary IgG concentrations of healthy horses ranged from 5.1 to 74.3 mg/L) and values of IgA ranging from 0.5 to 351 mg/L (in our study, the salivary IgA concentrations ranged from 7.2 to 38.5 mg/L) [17].

In serum, although to our knowledge, there are no other reports on horses to compare our results with, the values of IgG were higher than those for IgA, as has been described in other species [12].

In horses with EGUS, IgG and IgA showed a tendency to decrease in serum, but the results were not significant. To the author’s knowledge, there are no previous reports on horses regarding changes in IgG in gastrointestinal diseases or EGUS. However, our results would be in line with those reported in humans, where levels of serum IgG are significantly reduced in patients with Chron’s Disease and isolated ileal disease [26], and serum IgA showed a tendency to decrease in peptic ulcers [13]. One of the mechanisms proposed is that these patients have a stronger microbial dysbiosis, which may be a mechanism for inducing reduced serum IgG levels [26]. In addition, in human patients with gastric ulcers due to *H. pylori*, lower serum IgG has been associated with a predominance of the cell immune response, leading to increases in T lymphocytes and a decrease in the humoral immune response, leading to a decrease in B lymphocytes, which are the producers of IgG. This situation can be reversed after an appropriate treatment [27]. A similar process could occur in horses with EGGD, where CD3-positive cells, which correspond to T-cell lymphocytes, were more common than CD20-positive cells, which are B-cell lymphocytes in the mucosae of their stomachs [28].

In our report, in the saliva of horses with EGUS, IgG showed a similar tendency to decrease to that in serum. However, in saliva, the IgA concentrations had a different response and significantly increased in horses with EGUS. An increase in IgA in saliva in patients with gastric ulcers due *H. pillory* infection and also due to IBD has been described in humans [15,29]. Among the different possible causes that could be explored for these increases in IgA are changes in the oral microbiota or the influence of stress, since increases in IgA have been found in situations in which the sympathetic pathway is activated in other animal species and humans [16,30]. Along the same lines, increases in salivary alpha-amylase, which is a marker of sympathetic activation, have been found in the saliva of horses with EGUS compared to healthy horses [31]. In addition, a possible relationship with cellular immune system activation could be the cause of the increases in IgA found in the saliva in our study since the analyte that showed the higher significant correlation with IgA was ADA, which is related to the cellular immune function. IgA is part of the first line of defence of the immunological defence in epithelial tissues [32], and it could be postulated that the gastric mucosae, when suffering an injury, can produce IgA in the digestive tract locally, a fact that could contribute to their increase in saliva but not in serum.

Regarding the other biomarkers of the immune system that were measured in this report, ADA, S100A8-A9, and S100A12 showed higher values in the saliva of horses with EGUS than in healthy horses. In addition, ADA showed higher values in the EGUS compared to the non-EGUS group, especially in those with the glandular form. Increases in ADA in saliva of a similar magnitude have been previously reported in EGUS [31]. ADA is an enzyme related to T lymphoid system activity that could indicate the involvement of the immune system in the physiopathology of EGUS [25]. The two S100 proteins evaluated in this report showed increases in saliva in the EGUS group. S100A8-A9 showed increases of a similar magnitude to those found in our study, and similar increases have been previously reported in EGUS, likely reflecting an immune system activation [6]. On the other hand, S100 A12 increases in a variety of digestive disorders, including gastritis, inflammatory bowel disease (IBD), and Crohn’s disease, where it contributes to the recruitment and activation of immune cells [33]. Overall, the increase in S100s in the saliva of horses with ulcers, as observed in our study, could reflect an activation of the immune cells occurring in this disease. Regarding the biomarkers of redox status evaluated in this report, uric acid, FRAS, and AOPP showed increases in the saliva of EGUS. Increases in the glandular form of EGGD have been previously reported [11]. The increase in AOPP could indicate the existence of oxidative damage in this condition, which can lead to an increase in the antioxidant compounds such as uric acid and FRAS to compensate.

The correlations observed between salivary IgA and other immune biomarkers like ADA and calprotectin highlight the role of the mucosal immune response in EGUS’s pathophysiology. These biomarkers could potentially be used as an additional tool to monitor disease progression and response to treatment. Integrating these findings into diagnostic protocols could allow for earlier detection and improved monitoring of treatment efficacy in EGUS, enhancing overall clinical outcomes. As such, further studies should be carried out to evaluate if algorithms with the combination of these biomarkers could be useful for making clinical decisions. For example, an algorithm integrating various analytes that have a high sensitivity and specificity could be useful, since a value within the range of healthy horses could indicate that the horse is not likely to have EGUS at gastroscopy. In addition, a higher score in this algorithm for a horse with suspicions of EGUS would indicate a high probability of having EGUS at gastroscopy.

Serum values were not correlated with saliva in our study for any analyte. In addition, the analytes showed different behaviours in the serum of horses with EGUS compared to saliva. ADA and biomarkers of redox status did not show significant changes in serum, contrary to what occurred in saliva, where they increased. The lack of sensitivity of serum in the case of ADA and biomarkers of redox status for detecting EGUS has previously been reported [11]. In addition, S100 A8-A9 and acid uric showed significant decreases in the serum of horses with EGUS, whereas in saliva, they did increase, and the S100 A12 also showed a tendency to decrease in the serum of horses with EGUS. Although further studies should be carried out to elucidate the reasons for these divergences, in the case of S100 A8-A9 and S100-A12, lower values and reduced sensitivity for detecting diseases have been reported for serum compared to saliva in other species, such as pigs [34,35]. These differences may suggest the presence of distinct local and systemic immune responses. For instance, the elevated levels of IgA in saliva could be attributed to localized mucosal immune responses, which are often more sensitive to changes in gastrointestinal health compared to systemic responses reflected in serum [36]. Salivary IgA is primarily produced by plasma cells in mucosal tissues and serves as a first line of defence against tissue damage. Conversely, serum IgA is generated through a different pathway from the blood plasmatic cells being more related to systemic immunity, and may not exhibit the same responsiveness to local inflammatory processes [37]. This suggests that in the case of IgA, the mucosal immune system could be more dynamically engaged in response to the gastric disturbances characteristic of EGUS. Further research is needed to elucidate these mechanisms and assess the implications of such localized immune responses in the context of equine health.

IgA in the saliva of horses with EGUS also showed higher concentrations than in the saliva of horses with other diseases that were different to EGUS in our report. It is of interest to point out that EGUS can be associated with colic, a serious condition that has significant health risks and can lead to fatal outcomes in horses [38,39]. Further studies should be performed to evaluate the possible differences in salivary IgA and the other biomarkers in this report between horses with EGUS and colic in order to identify possible new biomarkers that could help to differentiate these two conditions.

This study has various limitations. While the assays for IgG and IgA have been validated in equine samples, there were no species-specific standards available for IgA. This limitation could potentially affect the accuracy of our results and their interpretation, as the differences in the cross-reactivity of heterologous antibodies made in humans and other species may yield misleading outcomes. To mitigate this issue, it would be of high importance to explore alternative approaches for further improving and validating the IgA assays. One possible option is the use of equine IgA standards obtained through the purification of equine IgA in the assay, which would enhance the reliability of our measurements. As such, it is of interest to point out that some procedures for purification previously described seem not to be practical for the saliva of horses [17]. A further limitation of the study is the relatively low number of animals used, given that the required sample size was estimated to be 39 horses per group, and there is also a possible bias in the use of different groups from different locations. Additionally, the effect of breed was not assessed in this study and further investigations should be made to clarify it. Overall, the results of this report should be considered to be preliminary, and further studies with a larger number of horses should be performed to confirm these findings and clarify the possible practical applications of the measurements of IgA in saliva as a biomarker in this disease. In addition, the possible application of this analyte for treatment monitoring of EGUS should be explored.

## 5. Conclusions

Based on the results of this manuscript, it can be concluded that IgG and IgA can be measured in horses’ saliva with the automated assays validated in this report. In addition, IgA significantly increased in the saliva of horses with EGUS compared to the healthy group. When compared to other biomarkers of immune system issues and oxidative stress, IgA in saliva showed a significant moderate correlation with ADA, indicating its possible involvement in the immune reaction occurring in EGUS. Further studies should be performed to elucidate the possible applications of IgA as a biomarker of EGUS.

## Figures and Tables

**Table 1:** Precision study of the immunoglobulin G and A assays in horse saliva.

Method	Comparison	Samples	Mean	SD	CV (%)	
IgG (mg/dL)	Intra-assay	High	8.45	0.42	4.98	
		Low	3.00	0.16	5.27	
	Inter-assay	High	9.06	0.57	6.27	
		Low	2.94	0.27	9.19	
IgA (mg/dL)	Intra-assay	High	8.06	0.24	2.99	
		Low	2.12	0.13	6.15	
	Inter-assay	High		7.74	0.56	7.23
		Low		2.28	0.23	10.00

SD: standard deviation, CV: coefficient of variation.

**Table 2:** Precision study of the immunoglobulin G and A assays in horse serum.

Method	Comparison	Samples	Mean	SD	CV (%)
IgG (mg/dL)	Intra-assay	High	845.00	33.95	4.02
		Low	310.20	9.12	2.94
	Inter-assay	High	803	27.52	3.43
		Low	335.6	32.35	9.64
IgA (mg/dL)	Intra-assay	High	7.05	0.22	3.11
		Low	1.18	0.08	7.09
	Inter-assay	High	7.56	0.58	7.63
		Low	1.7	0.19	11.00

SD: standard deviation, CV: coefficient of variation.

**Table 3:** Results of salivary analytes in healthy horses (n = 14), horses with EGGD and ESGD at the same time (EGGD + ESGD) (n = 25), horses with only EGGD (n = 25), horses with only ESGD (n = 21), and horses diagnosed with other gastrointestinal disorders apart from EGUS but with similar symptoms (non-EGUS, n = 20). Median values (interquartile range) are expressed. Statistical analysis: asterisks indicate significant differences between the different types of EGUS and non-EGUS groups, comparing with the healthy group using Dunn’s multiple comparisons post hoc test (*: *p* < 0.05; **: *p* < 0.01; ***: *p* < 0.001; ****: *p* < 0.0001); letters indicate significant results with the non-EGUS group (a: *p* < 0.001).

	EGGD + ESGD	EGGD	ESGD	Non-EGUS	Healthy
ADA (U/L)	196.8 **** (105.2–398.2)	156 **** (69.9–226.7)	125.2 **** (63.6–195.6)	45.8 (32.1–125.3)	20.6 (13.23–26.03)
Calprotectin (mg/L)	11.84 ** (8.64–35.36)	16.64 ** (2.88–24)	13.28 * (4.4–15.76)	15.36 *** (4.16–25.92)	1.6 (0.96–2.24)
S100A12 (ng/mL)	1.96 **** (1.86–2.17)	1.39 * (1.33–1.50)	1.38 * (1.34–1.52)	1.34 * (1.1–1.83)	0.58 (0.37–0.85)
Uric acid (µmol/L)	221 ****^a^ (112–331)	121 242 **** (74–193)	148 **** (68–300)	117 * (43–139)	50 (32–160)
FRAS (µmol/L)	860 ****^a^ (540–1270)	580 * (400–740)	540 ** (430–1120)	500 (240–630)	260 (208–373)
AOPP (µmol/L)	182.8 (123.6–383.4)	179.2 (90.4–304.8)	215.4 (114.8–350.7)	181.8 (67.9–512.5)	103.8 (78.59–147.3)

ADA: adenosine deaminase; FRAS: ferric-reducing ability of saliva; AOPP: advanced oxidation protein products; ESGD: Equine Squamous Gastric Disease; EGUS: Equine Gastric Ulcer Syndrome.

**Table 4:** Results of serum analytes in healthy horses (n = 14), horses with EGGD and ESGD at the same time (EGGD + ESGD) (n = 25), horses with only EGGD (n = 25), horses with only ESGD (n = 21), and horses diagnosed with other gastrointestinal disorders apart from EGUS but with similar symptoms (non-EGUS, n = 20). Median values (interquartile range) are expressed. Statistical analysis: asterisks indicate significant differences between the different types of EGUS and non-EGUS groups, comparing with the healthy group using Dunn’s multiple comparisons post hoc test (**: *p* < 0.01; ***: *p* < 0.001; ****: *p* < 0.0001). Letters indicate significant results with the non-EGUS group (a: *p* < 0.001).

	EGGD + ESGD	EGGD	ESGD	Non-EGUS	Healthy
ADA (U/L)	0.3 (0.2–0.42)	0.2 (0.1–0.2)	0.2 (0.1–0.3)	0.6 (0.5–0.7)	0.3 (0.2–0.42)
Calprotectin (mg/L)	0.05 **** (0.04–0.07)	0.04 **** (0.03–0.06)	0.05 *** (0.03–0.11)	0.05 *** (0.04–0.11)	0.17 ^a^ (0.15–0.2)
S100A12 (µg/mL)	0.50 (0.21–0.69)	0.45 (0.19–0.73)	0.49 (0.12–0.61)	0.64 (0.41–0.91)	0.75 (0.33–0.99)
Uric acid (µmol/L)	17 (13–22)	14 ** (9–19)	14 ** (9–19)	21 (11–30)	30 (17–33)
FRAP (µmol/L)	340 (290–390)	210 (110–330)	230 (80–380)	320 (210–450)	340 (290–390)
AOPP (µmol/L)	32.25 (25.9–39.08)	29.75 (25.28–44.85)	40.5 (27.5–49.1)	43.6 (31.1–65.2)	39.2 (34.5–43.6)

ADA: adenosine deaminase; FRAP: ferric-reducing ability of plasma; AOPP: advanced oxidation protein products; EGGD: equine glandular gastric disease; ESGD: Equine Squamous Gastric Disease; EGUS: Equine Gastric Ulcer Syndrome.

## Data Availability

The data presented in this study are available on request from the corresponding author.

## Supplementary Materials

The following supporting information can be downloaded at: https://www.mdpi.com/article/10.3390/biology13110891/s1, Table S1: Individual description and reason for gastroscopy of horses (n = 20) with a suspicious of Equine Gastric Ulcer Syndrome but with non-compatible gastroscopy images; Table S2: Description of the biomarkers’ correlations in equine saliva and serum.
